# Transcriptome analysis and prognosis of ALDH isoforms in human cancer

**DOI:** 10.1038/s41598-018-21123-4

**Published:** 2018-02-09

**Authors:** Peter Mu-Hsin Chang, Che-Hong Chen, Chi-Chun Yeh, Hsueh-Ju Lu, Tze-Tze Liu, Ming-Huang Chen, Chun-Yu Liu, Alexander T. H. Wu, Muh-Hwa Yang, Shyh-Kuan Tai, Daria Mochly-Rosen, Chi-Ying F. Huang

**Affiliations:** 1 Department of Oncology, Taipei Veterans General Hospital, Taipei, 112 Taiwan; 2 Faculty of Medicine, National Yang Ming University, Taipei, 112 Taiwan; 3 Department of Chemical and Systems Biology, Stanford University, School of Medicine, Stanford, CA 94305 USA; 4 Jin An Clinic, New Taipei City, 24256 Taiwan; 5 Division of Medical Oncology, Department of Internal Medicine, Chung Shan Medical University Hospital, Taichung, Taiwan; 6 School of Medicine, Chung Shan Medical University, Taichung, Taiwan; 7 Genome Research Center, National Yang-Ming University, Taipei, 112 Taiwan; 8 The Ph.D. Program for Translational Medicine, College of Science and Technology, Taipei Medical University, Taipei, 110 Taiwan; 9 Institute of Clinical Medicine, National Yang Ming University, Taipei, 112 Taiwan; 10 Division of Laryngology-Head and Neck Surgery, Taipei Veterans General Hospital, Taipei, 112 Taiwan; 11 Institute of Biopharmaceutical Sciences, National Yang Ming University, Taipei, 112 Taiwan

## Abstract

Overexpression of ALDH is associated with cancer stem-like features and poor cancer prognosis. High ALDH activity has been observed in cancer stem-like cells. There are a total of 19 human ALDH isoforms, all of which are associated with reducing oxidative stress and protecting cells from damage. However, it is unknown whether all ALDHs are associated with poor cancer prognosis and which ones play a significant role in cancer progression. In this study, we used RNA sequencing data from The Cancer Genome Atlas (TCGA) to evaluate the differential expression of 19 ALDH isoforms in 5 common human cancers. The 19 ALDH genes were analyzed with an integrating meta-analysis of cancer prognosis. Genotyping and next-generation RNA sequencing for 30 pairwise samples of head and neck squamous cell carcinoma were performed and compared with the TCGA cohort. The analysis showed that each ALDH isoform had a specific differential expression pattern, most of which were related to prognosis in human cancer. A lower expression of ALDH2 in the tumor was observed, which was independent from the ALDH2 rs671 SNP variant and the expression of other mitochondria-associated protein coding genes. This study provides new insight into the association between ALDH expression and cancer prognosis.

## Introduction

Carcinogenesis is an extremely complicated process that may involve multilevel mutations such as karyotype changes, loss of heterogeneity, DNA copy-number variations, sequence mutations and aberrant mRNA and/or protein expression. Among them, microarray and next-generation RNA sequencing (RNA-seq) have been widely used to identify oncogenic expression on a genome-wide scale because of the strength of simultaneous analysis of thousands of genes, which may help to identify novel biomarkers for treatment response, cancer prognosis and precision medicine^1^. High-throughput approaches for transcription-level changes of oncogenes, novel biomarkers and signaling can be identified for cancer phenotypes in different human cancers^2–4^. However, questions have been raised regarding the reproducibility and reliability of microarray experiments. The main challenge of these microarray studies are because of a small number of samples, inconsistent tissue sample quality from the DNA/RNA extraction and incomprehensive clinical data for analysis. In recent years, The Cancer Genome Atlas (TCGA: https://cancergenome.nih.gov/) program, which includes comprehensive, multi-dimensional maps of the key genomic changes in more than 30 types of cancer, has been used for the cancer studies. The TCGA dataset places an emphasis on the tissue sample quality that was used and has more than 2.5 petabytes of data, including pairwise tumor/normal tissues, from more than 10,000 patients. It has become one of the most powerful and popular tools for genomic studies of human cancers^5–8^.

In human, the multigene ALDH family that consists of 19 different isozymes has been identified due to similar amino acid sequences and functions^9,10^. Furthermore, elevated ALDH activity has been used as a cancer stem cell biomarker^11^. Cancer stem-like features account for the relative aggressiveness of tumors and are potential prognostic indicators for patients with cancer^12^. Several recent studies have shown that ALDH1A1 and ALDH3A1 may detoxify cyclophosphamide and result in cancer resistance^13,14^. High ALDH1A3 expression has been reported as a poor prognostic marker for breast cancer and cholangiocarcinoma^10,15^. However, ALDHs are also well known for metabolizing aldehydes and thus reducing the oxidative stress in cells from damage. For example, ALDH2 has the lowest Michaelis constant for acetaldehyde, which has been classified as a group 1 carcinogen by the International Agency for Research on Cancer^16^. Reduction in ALDH2 activity increases acetaldehyde accumulation in the human body, which increases the cancer risk in patients, especially in those that consume alcohol^17^. Finally, the exact functions of other ALDH isoforms remain unclear; therefore, a more comprehensive approach for the differential expressions (DEs) and prognosis of all ALDH isoforms in human cancers is warranted.

*In silico* analysis has been commonly utilized in genomic studies, thus resulting in public microarray or RNA-seq datasets^1,18^. These high-throughput bioinformatics tools can provide insight into the biological dynamics and functional validation of candidate genes. The challenge of reproducibility for an individual microarray study may potentially be improved by a systematic approach using standardized methods^19,20^. Prognoscan (http://www.abren.net/PrognoScan/) is a bioinformatics tool that contains more than 70 microarray studies from 13 different human cancer types with clinical prognosis^21^. It has been used widely for human cancer research^22–25^ and provides a method to cross-link a group of candidate genes with prognoses in a systematic manner. In this study, we analyzed the DEs of all 19 ALDH isotypes using the TCGA RNA-seq dataset and integrated prognostic evaluations from the Prognoscan microarray meta-analysis. Finally, the 30 pairwise head and neck squamous cell carcinomas (HNSCs) from Taiwanese patients were used to compare the ALDH2 genotype with the DEs and cancer prognosis.

## Results

### Various differential expressions exist in the 19 ALDH isoforms compared to the TCGA cohort

**Figure 1:**
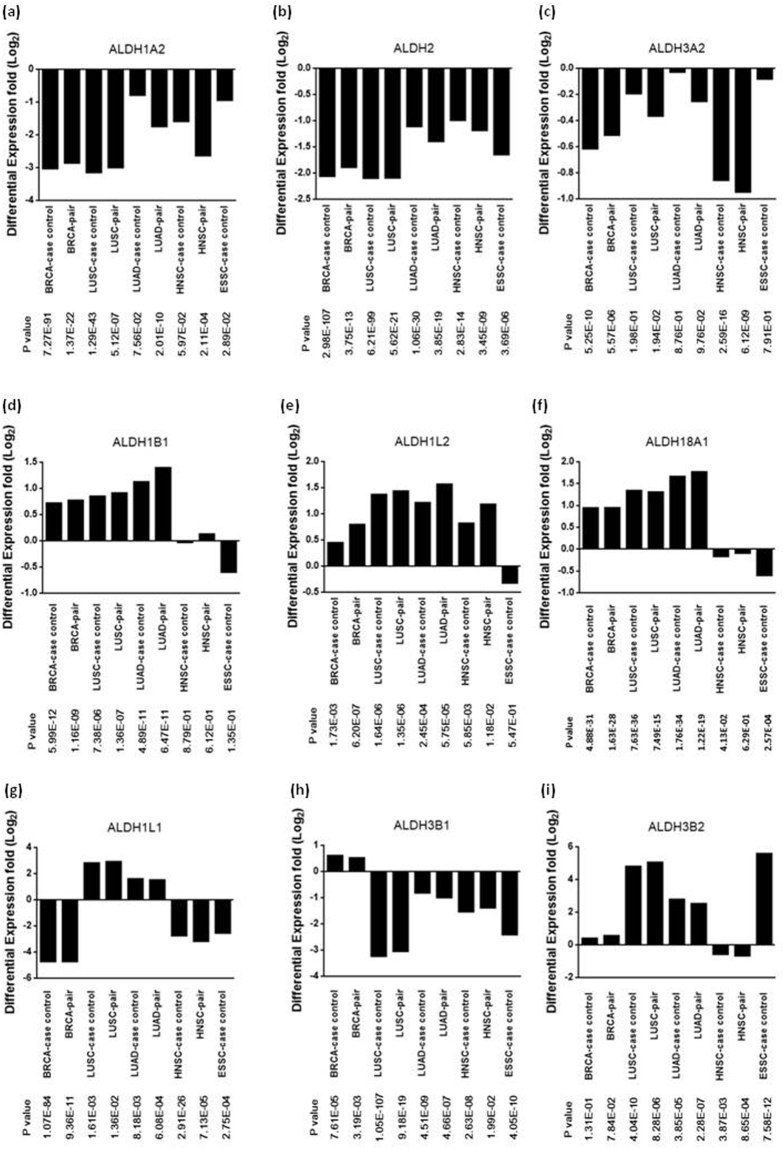
Differential expression of ALDH isoforms in five cancer types. (**a**) ALDH1A2; (**b**) ALDH2; (**c**) ALDH3A2; (**d**) ALDH1B1; (**e**) ALDH1L2; (**f**) ALDH18A1; (**g**) ALDH1L1; (**h**) ALDH3B1; (**i**) ALDH3B2. Columns from left to right: BRCA case-control, BRCA pair, LUSC case-control, LUSC pair, LUAD case-control, LUAD pair, HNSC case-control, HNSC pair. ESSC case-control. Ratio was shown in Log_2_ transformation. BRCA: breast cancer; LUSC: lung squamous cell carcinoma; LUAD: lung adenocarcinoma; HNSC: head and neck squamous cell carcinoma; ESSC: esophageal squamous cell carcinoma; case-control (all tumors vs. normal tissue); pair (pairwise tumor vs. normal tissue); *Only one pair of ESSC tumor vs. normal so there is only case-control comparison of ESSC data.

From the TCGA database, the RNA-seq data were extracted for samples of breast cancer (BRCA) (1097 tumor vs. 114 normal samples), lung adenocarcinoma (LUAD) (515 tumor vs. 59 normal samples), lung squamous cell carcinoma (LUSC) (502 tumor vs. 51 normal samples), esophageal squamous cell carcinoma (ESSC) (82 tumor vs. 8 normal samples) and HNSC (520 tumor vs. 44 normal samples). The DEs for all 19 ALDH tumors vs. normal samples in 5 cancer types are shown in Fig. 1 and Supplemental Fig. 1. Generally, the pairwise comparison (BRCA, 114 pairs; LUAD, 59 pairs; LUSC, 51 pairs; HNSC, 44 pairs) showed a similar trend with case-control comparison, while the pairwise study showed a more significant p-value than the case-control study. We hypothesized that pairwise samples have more specific DEs because individual heterogeneity was minimized. Interestingly, there were several different DEs among the 19 ALDH isoforms. For example, ALDH1A2, ALDH2, ALDH3A2 and ALDH9A1 were downregulated in all tumors among the 5 cancer types (Fig. 1a,b,c and Supplemental Fig. 1a), whereas ALDH1B1, ALDH1L2 and ALDH18A1 were most upregulated in tumor parts (Fig. 1d,e,f). Some tumor type-specific DEs were observed for ALDH1L1, ALDH3B1, ALDH3B2, ALDH4A1 and ALDH7A1 (Fig. 1g,h,i and Supplemental Fig. 1b,c). For validation with other non-TCGA cohorts, we also use the NGS study from Djureinovic *et al*., both including LUSC and LUAD to compare with normal tissue of lung (GSE81089). In addition, tumor vs. normal microarray profiles for HNSC (GSE6631) and BRCA (GSE25291) have also been downloaded and analyzed. The DEs showed similar trends comparing with TCGA cohort (Supplemental Fig. 2). This result suggested that, at least among these 5 common cancer types, there were various DEs for each individual ALDH isoform.

### ALDH differential expression is associated with prognosis in human cancer

**Figure 2:**
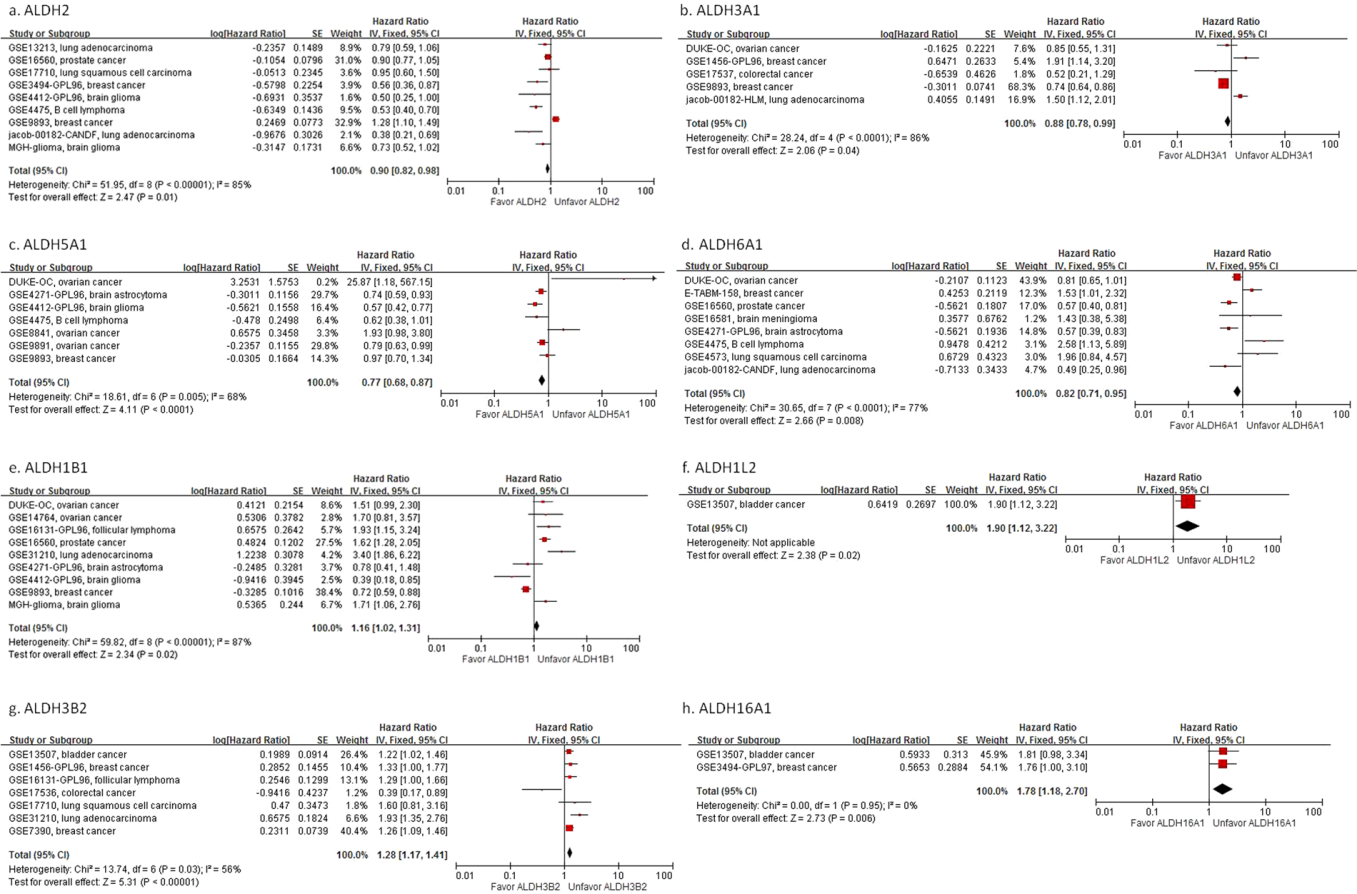
Meta-analysis of overall survival for different ALDH isoforms. “Favor” indicates expression of candidate ALDH toward better a prognosis, whereas “Unfavor” indicates expression toward poorer prognosis. Lower expression of ALDH2, ALDH3A1, ALDH5A1 and ALDH6A1 but higher expression of ALDH1B1, ALDH1L2, ALDH3B2 and ALDH16A1 in tumors was associated with poorer overall survival. (**a**) ALDH2; (**b**) ALDH3A1; (**c**) ALDH5A1; (**d**) ALDH6A1; (**e**) ALDH1B1; (**f**) ALDH1L2; (**g**) ALDH3B2; (**h**) ALDH16A1.

**Figure 3:**
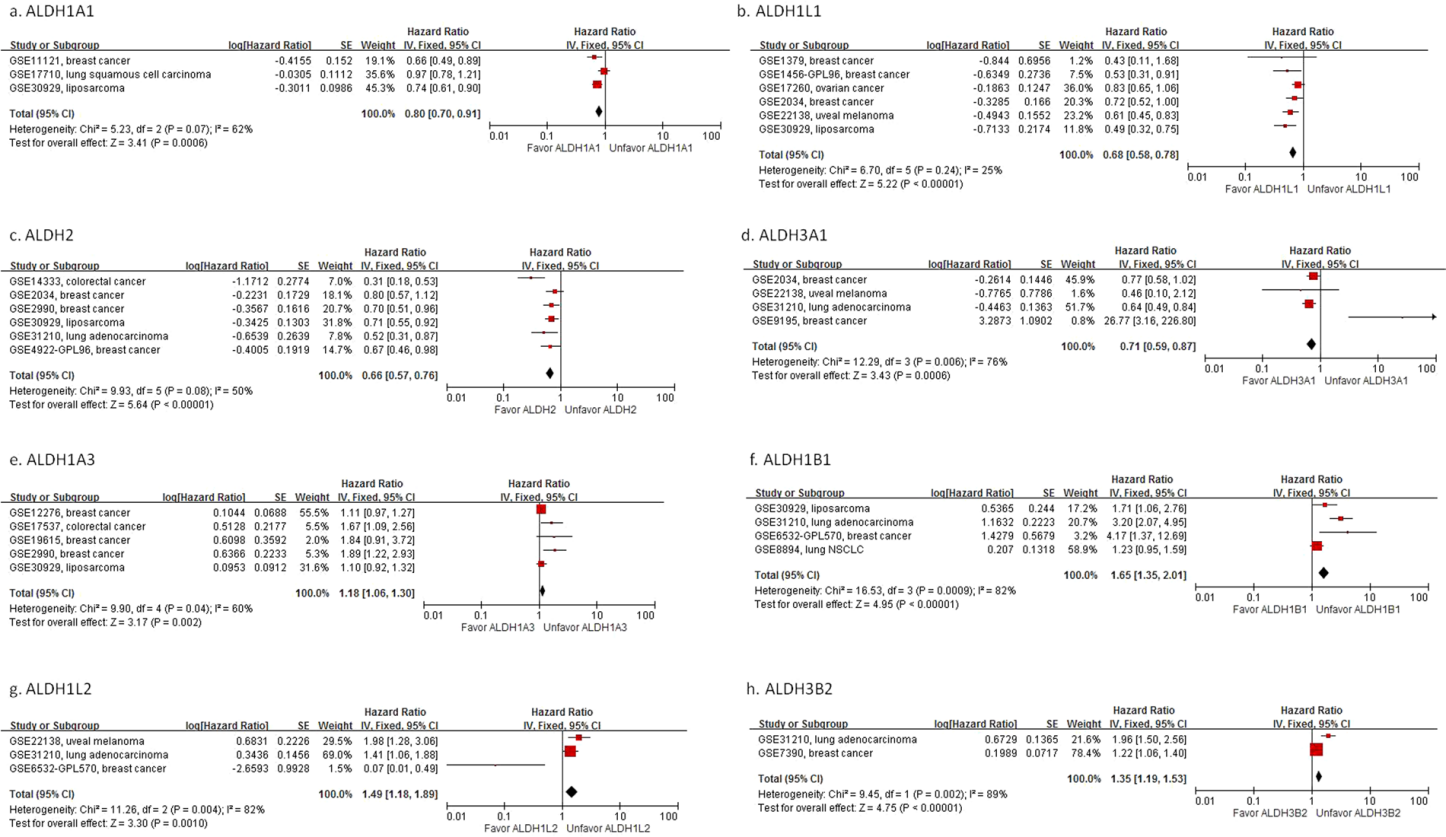
Meta-analysis of progression-free survival for different ALDH isoforms. “Favor” indicates expression of candidate ALDH toward better prognosis, whereas “Unfavor” indicates expression toward poorer prognosis. Lower expression of ALDH1A1, ALDH1L1, ALDH2, ALDH3A1, ALDH3A2, ALDH3B1, ALDH5A1, ALDH6A1 and ALDH9A1, whereas higher expression of ALDH1A3, ALDH1B1, ALDH1L2, ALDH3B2, ALDH8A1 and ALDH18A1 was associated with poorer progression free survival. (**a**) ALDH2; (**b**) ALDH3A1; (**c**) ALDH5A1; (**d**) ALDH6A1; (**e**) ALDH1B1; (**f**) ALDH1L2; (**g**) ALDH3B2; (**h**) ALDH8A1.

We applied all 19 ALDH isoforms into Prognoscan to evaluate the survival differences among high and low expressing subgroups. The microarray studies with corrected p-values < 0.05 were extracted from the querying result of each isozyme and meta-analyses were performed individually. As shown in Figs 2 and 3, various prognostic values were observed among the ALDH isoforms that had lower expressions of ALDH2, ALDH3A1, ALDH5A1 and ALDH6A1 (Fig. 2a–d) but higher expressions of ALDH1B1, ALDH1L2, ALDH3B2 and ALDH16A1 (Fig. 2e–h) in tumors that were associated with poorer OS. Lower expression of ALDH1A1, ALDH1L1, ALDH2, ALDH3A1, ALDH3A2, ALDH3B1, ALDH5A1, ALDH6A1 and ALDH9A1 (Fig. 3a–d, Supplemental Fig. 3a–e) while higher expression of ALDH1A3, ALDH1B1, ALDH1L2, ALDH3B2, ALDH8A1 and ALDH18A1 (Fig. 3e–h, Supplemental Fig. 3f and g) were associated with poorer Progression-free survival (PFS). Interestingly, these prognostic trends were compatible with the DEs of most ALDH isoforms (Supplemental Fig. 4), which implied that ALDH DEs were associated with cancer prognosis.

### Lower ALDH2 expression is observed in tumors and is associated with poor cancer prognosis

**Figure 4:**
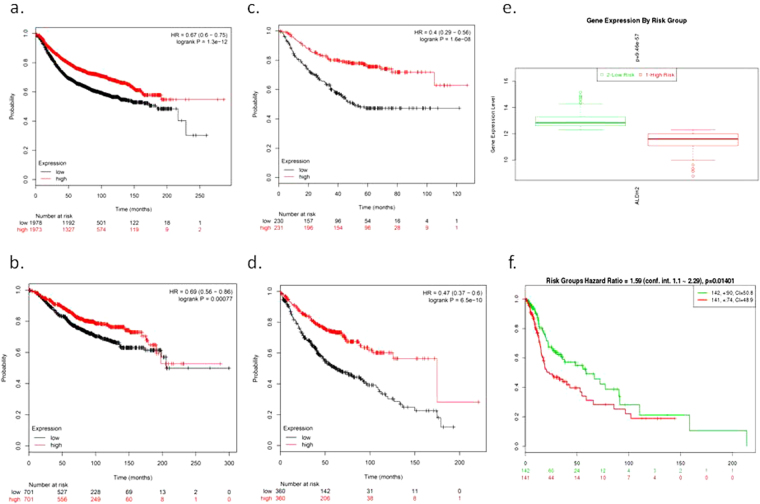
Survival curves of high vs. low ALDH2 expression in patients with cancer. (**a**) Kaplan-Meier relapse-free survival for patients with breast cancer. (**b**) Kaplan-Meier overall survival for patients with breast cancer. (**c**) Kaplan-Meier time-to-progression for patients with lung adenocarcinoma. (**d**) Kaplan-Meier overall survival for patients with lung adenocarcinoma. Red: High; Black: Low. HR: Hazard ratio. (**e**) The high expression of ALDH2 was identified as “low-risk” (green), while low expression of ALDH2 as “high-risk” (red). (**f**) Kaplan-Meier overall survival for patients with head and neck squamous cell carcinoma. Green: High; Red: Low.

Since ALDH2 is the most well-known ALDH isozyme for its function to reduce cancer risk^26^, we were especially interested when we observed that ALDH2 expression in tumors was downregulated and associated with poor cancer prognosis. First, we evaluated the DE for ALDH2 in our VGHTPE cohort. Fifteen tumor samples and 3 normal samples were within the QC criteria. Contrary to the commonly observed EGFR overexpression in HNSC, there were similar ALDH2 downregulations in both case-control (15 tumors vs. 3 normal) and pairwise (3 tumors vs. 3 normal) comparisons, although without a significant p-value, which may be due to the small number of samples (Supplemental Table 1). To further validate the prognostic role of the DE observed for ALDH2 in human cancer, we used KM plotter (http://kmplot.com/analysis/), which collected 10,188 human cancer microarray samples and normalized them together to generate a common high vs. low comparison of the DEs for each evaluated gene for the indicated cancer^27^. As shown in Fig. 4, lower ALDH2 expression in the tumor was also associated with significantly poorer prognosis in BRCA (RFS for 1973 high vs. 1978 low samples, HR = 0.67, CI = 0.6–0.75, p-value = 0; OS for high 701 low vs. 701 low samples, HR = 0.69; CI = 0.56–0.86, p-value = 0.0008) (Fig. 4a and b) and LUAD (time to first progression [FP] for 231 high vs. 230 low samples, HR = 0.4, CI = 0.29–0.56, p-value = 0; OS for 360 high vs. 360 low samples, HR = 0.47, CI = 0.7–0.6, p-value = 0) (Fig. 4c and d). Because there is no HNSC profile in KM plotter and only one HNSC study in Prognoscan with small case number (N = 28), we used SurvExpress (http://bioinformatica.mty.itesm.mx:8080/Biomatec/SurvivaX.jsp)^28^ to extract survival from HNSC TCGA cohort. The analysis showed that ALDH2 high expressed patients (N = 142) had significantly better survival than ALDH2 low expressed patients (N = 141) (HR = 1.59, CI = 1.1–2.29, p-value = 0.014) (Fig. 4e and f).

### ALDH2 expression in the tumor is independent from ALDH2*2 SNP and other mitochondria-associated proteins

**Figure 5:**
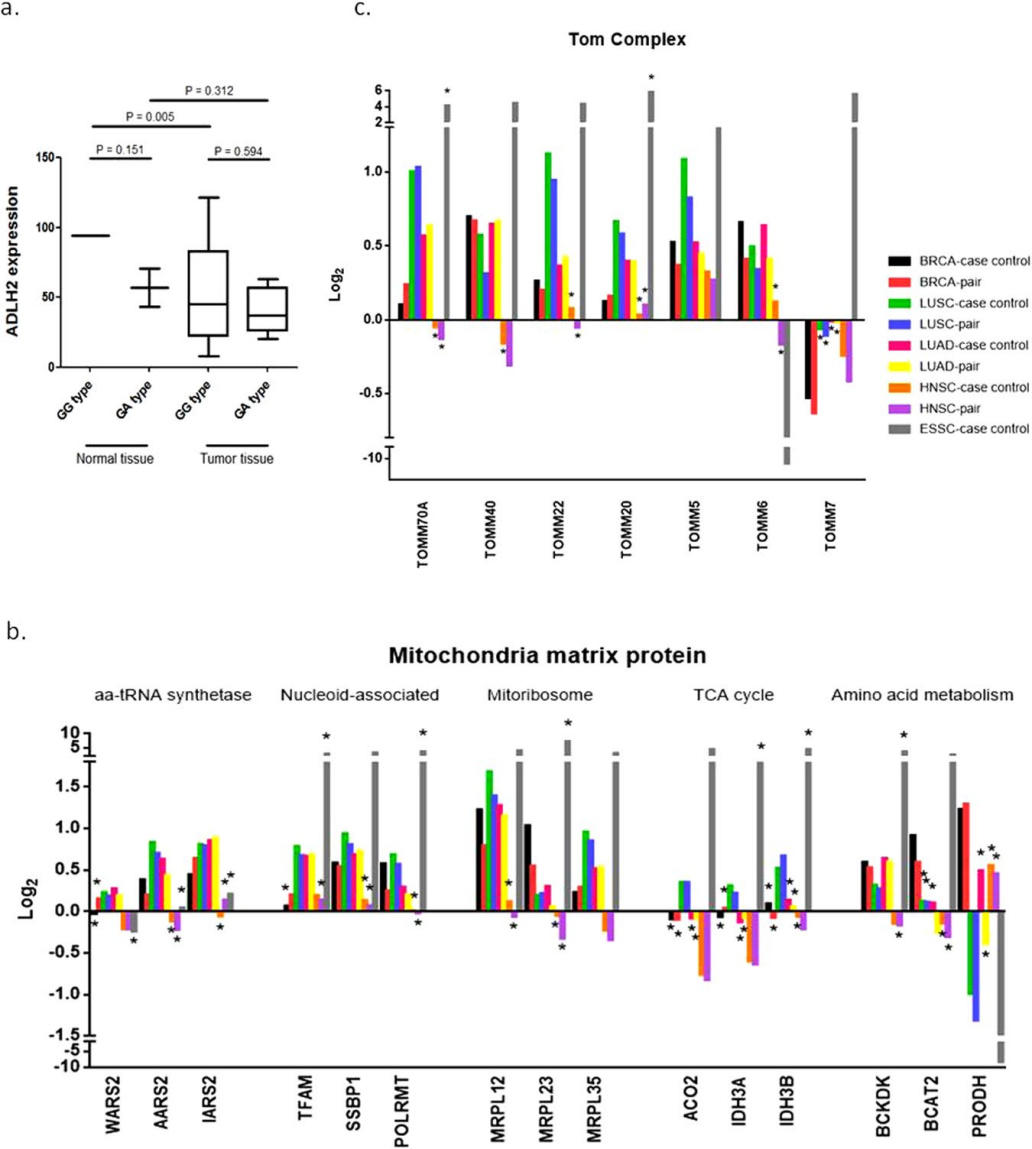
Correlation with ALDH genotyping, expression and other mitochondria-associated proteins. (**a**) Downregulation of ALDH2 is generally observed between wild types (rs671 GG) and heterozygotes (rs671 GA). Data from the Taipei Veteran’s General Hospital cohort. Y-axis denotes the reads per kilobase of exon model per million mapped reads (RPKM) value. (**b**) Expression of candidate genes (baseline) with functional group annotations (top) for mitochondria matrix proteins are shown among the 5 cancers. (**c**) Mitochondrial TOM complex. BRCA: breast cancer, LUSC: lung squamous cell carcinoma, LUAD: lung adenocarcinoma, HNSC: head and neck squamous cell carcinoma, ESSC: esophageal squamous cell carcinoma, case-control: all tumor vs. normal, pair: pairwise tumor vs. normal. All p-values > 0.05 were marked with an asterisk (*).

Since the ALDH2 rs671 SNP is specifically common in the Asian and Taiwanese populations^16^, we performed genotyping for HNSC patient samples and also compared the RNA expression between the tumor and normal tissues. Interestingly, comparisons of ALDH2 genotype and expression levels in samples derived from our VGHTPE cohort resulted in the identification of a similar trend of lower ALDH2 expression in tumor samples both in the ALDH2 rs671 GG wild type allele and in the GA heterozygous allele when compared to normal tissues (Fig. 5a). Furthermore, since ALDH2 enzyme only exists as an active form in the mitochondrial matrix, we thus compared the DEs of functional coding genes in mitochondrial matrix to see whether down-regulation of ALDH2 in tumor is independent from other mitochondria matrix associating proteins. As shown in Fig. 5b, most mitochondrial matrix protein-coding genes were significantly upregulated in tumors between the 5 different cancers, which was opposite to the ALDH2 DE. In addition, TOM complex accounts for transporting functional proteins into mitochondria and is upregulated in some cancer cells to stabilize anti-apoptotic proteins^29,30^. Therefore, we also compared the DEs of TOM complex genes to see whether TOM complex genes are associated with ALDH2 expression. As shown in Fig. 5c, most TOM complex proteins were upregulated in tumors among the 5 cancer types, which is also opposite to the DE of ALDH2.

## Discussion

In this study, we used an integrated analysis to evaluate the DEs for all ALDH isotypes as well as their correlation with cancer prognosis. We found that there were different DEs and prognosis among the 19 ALDH subtypes, suggesting that they may have individual functional roles in cancer prognosis. Interestingly, we found that some ALDHs are downregulated in tumors and are also associated with poorer prognosis, especially for ALDH2. This result is inconsistent with the current hypothesis for high ALDH activity in tumors with cancer stem-like features. ALDH2 is regarded as a mitochondrial enzyme with an activated form existing only in the mitochondrial matrix. In addition to acetaldehyde metabolism, it also plays a role in the removal of other reactive aldehydes derived from oxidative stress and lipid peroxidation, such as 4-hydroxy-nonenal and malondialdehyde. In human hepatocellular carcinoma, the downregulation of ALDH2 in the tumor has also been reported^31^. On the other hand, increasing mitochondria-associated gene expression is commonly observed during carcinogenesis or cancer progression^32,33^, which was compatible but also opposite to the ALDH2 downregulation in the current study. These results all suggested that downregulation of ALDH2 in tumor may be associated with cancer progression and influence prognosis. For the proof of concept, we used the ALDH2 agonist, Alda-1, which can specifically enhance enzymatic activity both in ALDH2 wild type and mutant form^34^ to treat cancer cells and observe the responsive phenotypes. The preliminary results showed that Alda-1 inhibited the migration/invasion ability as well as the cell viability in aggressive breast cancer MDA-MB-468 and MDA-MB-231 cells (Supplemental Fig. 5a and b). On the contrary, using siRNA to knockdown ALDH2 in MDA-MB-468 also increased migration (Supplemental Fig. 5e). In addition, both glycolysis and mitochondria respiration of HNSC FaDu cell was downregulated after Alda-1 treatment (Supplemental Fig. 5c and d). These results suggest that ALDH2 activity may be associated with cancer metabolism and influence cancer progression. Therefore, further exploratory experiments to confirm the underlying mechanism are warranted.

The East Asian-specific ALDH2 rs671 SNP has raised attention and has been demonstrated to be a strong genetic factor for increased cancer risk, especially in patients with high alcohol intake^16^. ALDH2 rs671 is a SNP resulting in a K487E mutation^35^. This single amino acid mutation causes a severe functional deficiency of the ALDH2 enzymatic activity which then leads to acetaldehyde accumulation, even after intake of a single alcoholic beverage^17^ and is believed to be the underlying cause of increased cancer risks for HNSC^36^ and ESSC^17^. In the current study, because the TCGA cohort represents data for mostly non-Asian subjects, the effects of the ALDH2 rs671 SNP on the DE for ALDH2 could only be analyzed from our own VGHTPE cohort. The results showed similar downregulation of ALDH2 in tumors with the ALDH2 GG wild type allele and the rs671 GA heterozygous allele, suggesting that this may be a general regulation independent from the ALDH2 SNP. Larger data collection from cohorts of Asian patients with cancer is therefore needed for future studies.

Furthermore, some tumor type-specific DEs were also observed. We noticed that more variations existed in HNSC when compared to the other 4 cancer types. HNSC is the most common cancer occurrence among middle-aged males in Taiwan and the sixth most common cancer in the world^37^. The etiology of HNSC is attributed to the exposure to environmental carcinogens derived from alcoholic beverages, cigarette smoke and betel nut use. Exposure to these environmental carcinogens incurs repeated damage to the upper aerodigestive tract mucosa cells and results in DNA damage, inappropriate modulation of autophagy and and carcinogenesis^38–40^. The complex interaction of several environmental carcinogens makes the spectrum of the mutations observed in HNSCs very heterogeneous and individualized. From the PCA analyses of both the TCGA database and our VGHTPE cohorts, it is almost impossible to identify a simple differentiating genetic signature based on comparisons between the tumor vs. normal tissues from patients with HNSC (Supplemental Fig. 6). Compared to other cancers with more separated gene expression between the tumor and normal tissues, this phenomenon may account for the controversial result between HNSC and the other 4 cancers.

Lastly, there are some limitations in this study. First, the retrospective cohort from our own databank is small and with an inevitable selection bias. Furthermore, the mitochondrial DNA (mtDNA) was not used for the RNA-seq, which also makes it impossible to evaluate their DEs with the DEs for other nuclear DNA coding mitochondria proteins. Finally, only 5 common cancer types were involved in this pilot study; therefore, any specific variations between cancers should be concluded only after completing a more comprehensive analysis with even more cancer types. For the prognostic survey of more other cancers, a newly developed bioinformatics tool “SurvExpress” (http://bioinformatica.mty.itesm.mx:8080/Biomatec/SurvivaX.jsp), which contains more 20,000 patient samples from 142 datasets, can be used for cross validation and more comprehensive analysis with multiple genes could be achieved in the future^28^.

In summary, this study provides a new insight into the ALDH family with their DEs in tumors vs. normal tissues as well as their association with cancer prognosis. For the high prevalence of the ALDH2 rs671 SNP, ALDH2 downregulation not only increases cancer risk but also influences cancer prognosis. This study provided the first systemic analysis for the differential expression and prognosis of all 19 human ALDH isoenzymes from publically available datasets, which may be applicable for other functional group of oncogenes, such as HER or VEGF families. Novel ALDH modulators could also be developed in the future, according to the prognostic role of each ALDH isoform as the biomarker. The results may have significant clinical implications and may also raise concerns for public health issues. Further research is therefore needed to focus on the relationship between the ALDH2 SNP, DE and their associated cancer phenotypes.

## Methods

### Gene expression profiling and differential gene expression analysis from TCGA

Using the TCGA database^41^, we extracted expression values of protein-coding genes for the following five types of carcinomas: LUAD, LUSC, BRCA, ESSC and HNSC. The expression values of protein-coding genes for adjacent non-cancerous tissues from surgical specimens were also extracted. The expression value data were extracted from the TCGA data matrix in August 2015 with the following criteria: disease type: (LUSC, LUAD, BRCA and HNSC), data type: RNASeq V2, data level: 3, batch number: all, platform: UNC (IlluminaHiSeq_RNASeqV2). ESSC data was extracted additionally in July 2017. The fold change of expression values between cancerous and normal tissues were expressed as log_2_ transformation. The gene-specific read counts were preprocessed with quantile normalization with the R package preprocessCore. The calculated p-values were adjusted to q-values for multiple testing using the Benjamini–Hochberg correction.

### In silico analysis of prognosis and ALDH expression in human cancer

PrognoScan is a bioinformatics tool that identifies an optimal threshold with the minimum p-value to separate the “high” and “low” expressing groups for survival difference in the selected genes. To control for type I errors, the p-value was corrected by the standard formula and shown as “corrected p-value”^21^. First, we extracted the microarray studies from only solid tumors with corrected p-values < 0.05, which meant the prognosis of the high vs. low expressing subgroups could be separated significantly. Hazard ratios (HRs) and 95% confidence intervals (CIs) for overall survival (OS) and PFS of each selected microarray study were downloaded from the Prognoscan database. The survival results were pooled with the meta-analysis through the Review Manager software, version 5.3 (Cochrane Collaboration).

### Analysis of HNSC clinical samples and data collection

Thirty matched, pairwise tumor/normal human HNSC samples, which were stored in liquid nitrogen immediately after resection, were selected from collections in the Taipei Veteran’s General Hospital (VGHTPE) tissue bank. All participants have signed informed consents before donating their tissue samples into this legal tissue bank. Also available with the 30 matched pairwise samples were confidential clinicopathological data that were used for the genomic study and correlation analysis in this study. DNA was extracted from these samples and dissolved in 1 x TE buffer (10 mM Tris, 1 mM EDTA, pH 8.0) for ALDH2 SNP determination. The QC of DNA OD was within 1.8–2.0. The quantified samples were then diluted to 10–20 ng/µl. Total RNA (1–5 µg) with concentrations >200 ng/μl were decontaminated by using DNase I and dissolved in 1 x TE or RNase-free H_2_O. The QC of RNA for the RNA integrity number (RIN) was within 8.0 and the OD 260/280 was within 1.8–2.0. The extracted samples were then transferred in dry ice packages to the Yang Ming National University Genomic Center for further genotyping and RNA-seq. The local ethics committee (Taipei Veterans General Hospital, Taiwan, R.O.C.) approved this study (TPEVGH IRB No.: 2015–08–003CC). All experiments were performed in accordance with relevant guidelines and regulations.

### Genotyping and whole transcriptome sequencing for HNSC

Genotyping of ALDH2*2 (rs671) was performed by Sequenom MassARRAY technology with iPLEX gold chemistry (Sequenom, San Diego, CA, USA). Briefly, the PCR primers and single-base extension primers were designed using the Assay Design Suite v2.0 software. The genotyping analysis was performed using the iPLEX Gold Reagent Kit (Sequenom) according to the manufacturer’s instruction. PCR followed by single-base primer extension was performed with 10 μg of the DNA sample (10 ng/µl). The extended reaction products were purified by cation-exchange resins and then spotted onto a 384-format SpectroCHIP II array using a MassArray Nanodispenser RS1000. Mass determination was performed on a MassARRAY Compact Analyzer. The resulting spectra were processed and alleles called with the MassARRAY Typer 4.0 (Sequenom) using the default settings. Extraction of RNA from frozen tissue samples was performed using the Qiagen RNeasyMini Kit. The quality of the RNA was assessed using the Agilent 2100 Bioanalyzer (Agilent Technologies, Santa Clara, CA, USA); samples with RIN >8 were used for further whole genome RNA-seq. The directional RNA-seq libraries were prepared using TruSeq Stranded mRNA Sample Prep Kit (Illumina). The sequencing libraries were sequenced on the HiSeq. 2500 platform (Illumina, San Diego, CA) by single-end sequencing with 100 bp read lengths to a depth of 28 to 42 million reads for each library. The RNA-seq data was analyzed with the CLC genomics workbench (Qiagen, Hilden, Germany). The quality of the raw read data in FASTQ format was assessed and reads of low quality were trimmed or removed. The adapter sequences were trimmed also. The sequenced reads were aligned to the NCBI_GRCH38 human reference genome and, following the removal of multi-mapping reads, converted to gene-specific read counts for annotated genes in the form of a reads per kilobase of exon model per million mapped reads (RPKM) value. The gene-specific read counts of HNSC samples were preprocessed with quantile normalization with the R package preprocessCore, with the sample procedure as processing TCGA data.

### Cell viability assay

MDA-MB-468 and MDA-MB-231 Cells (1 × 10^4^) were seeded onto 24-well plates for 24 h and then treated with indicated concentration of Alda-1 (A kind gift from Dr. Che-Hong Chen, Stanford University) for 72 h. The treated cells were added 0.5 mg/mL MTT (Sigma-Aldrich) to each well and incubated for 3 h at 37 °C. The violet MTT formazan precipitates were subsequently dissolved in 100 μL of DMSO. The absorbance at 570 nm was measured on an UQuant reader.

### Migration and invasion assays

The migration and invasion assays were performed in 24-well plate for 12 and 20 hours respectively. MDA-MB-468 cells (5 × 10^4^) in 200 μL of serum free medium were seeded onto upper Cell Culture Insert with 8 μm pores (Greiner Bio One) for migration assay and Matrigel matrix (Corning) coated Cell Culture Insert for invasion assay. The lower chamber contained 900 μL of complete medium. The cells migrated or invaded to the Cell Culture Insert membrane which were fixed with methanol for 10 minutes and stained with 0.005% crystal violet for 1 hour. The numbers of migrated or invaded cells were counted under the microscope from 10 random fields. For silencing ALDH2 assay, MDA-MB-468 were seeded for 24 hours and transfected with control siRNA or siALDH2 using Dharmafect 1 transfection reagent according to the manufacturer’s protocol. (Dharmacon, CO, USA) After 48 hours of transfection, cells were collected and resuspended in serum free medium for migration assay.

### Seahorse metabolism assay

2 × 10^4^ FaDu Cells were seeded in XF24 cell culture microplates, with adding 10 μM Alda-1 or not and activated the probe in non CO2 incubator on the first day. Second day, replacing growth medium with assay medium in XF24 cell culture microplates at least for 1 hour at 37 °C before running the assay. Next, oligomycin, FCCP and Rotenone/antimycine A were loaded in sensor cartridge and then sensor cartridge was set in XF24 analyzer to correct the condition. After the correction, began to metabolic determination.

### Statistical analysis

Overall pooled hazard ratios (HRs) were analyzed with a fixed effect model. Heterogeneity between microarray studies was investigated using Chi-square tests and the *I*^2^ index that expresses the percentage variability of the results related to the heterogeneity rather than to the sampling error. Statistical significance of the overall result was expressed with the probability value (p-value) in the “test for overall effect.” The result is regarded as statistically significant if p < 0.05. We compared the expression values of the 19 ALDH isotypes in normal and cancerous tissues and used the Mann-Whitney *U* test to determine the level of statistical significance for the differences in expression values. To determine the significance of differential gene expression between cancerous and normal samples in case-control comparisons, cancerous and normal samples were treated as independent samples and the two-sample test was used. For the pair-wised comparison, cancer and normal tissue samples from the same patient were treated as dependent samples and the paired difference test was used. We used the expression values of all protein-coding genes to depict the level of similarity between the cancer and normal tissue sample via the principal coordinate analysis (PCA) plot. Pearson correlations were used to approximate distances between samples in the PCA plot. Clinicopathological variables were compared using the Chi-square test or the Fisher’s exact test to differentiate between each other. A p-value of <0.05 was regarded as statistically significant in the 2-sided tests. Kaplan-Meier methods were used to evaluate PFS or OS. Log-rank tests were used for comparisons. All statistical analyses were performed using the SPSS statistical software version 18 (SPSS, Chicago, IL, USA) and R package (version 3.01, http://www.rproject.Org).

### Availability of data and materials

The datasets generated and/or analyzed during the current study are available in the TCGA repository (http://cancergenome.nih.gov/), PrognoScan repository (http://www.abren.net/PrognoScan/), KM plotter repository (http://kmplot.com/analysis/index) and SurvExpress repository (http://bioinformatica.mty.itesm.mx).

## Electronic supplementary material

Supplementary Information
