# Supplementary material for: Transcriptome analysis and prognosis of ALDH isoforms in human cancer
Source: Sci Rep. 2018 Feb 9;8:2713. doi: 10.1038/s41598-018-21123-4 (PMC5807355; doi:10.1038/s41598-018-21123-4)
Supplement: Supplementary file 1 — Supplementary Information [file 41598_2018_21123_MOESM1_ESM.pdf]

Transcriptome analysis and prognosis of ALDH isoforms in human cancer

Peter Mu-Hsin Chang, Che-Hong Chen, Chi-Chun Yeh, Hsueh-Ju Lu, Tze-Tze Liu, Ming-Huang Chen, Chun-Yu Liu,

Alexander TH Wu, Muh-Hwa Yang, Shyh-Kuan Tai, Daria Mochly-Rosen, Chi-Ying F. Huang

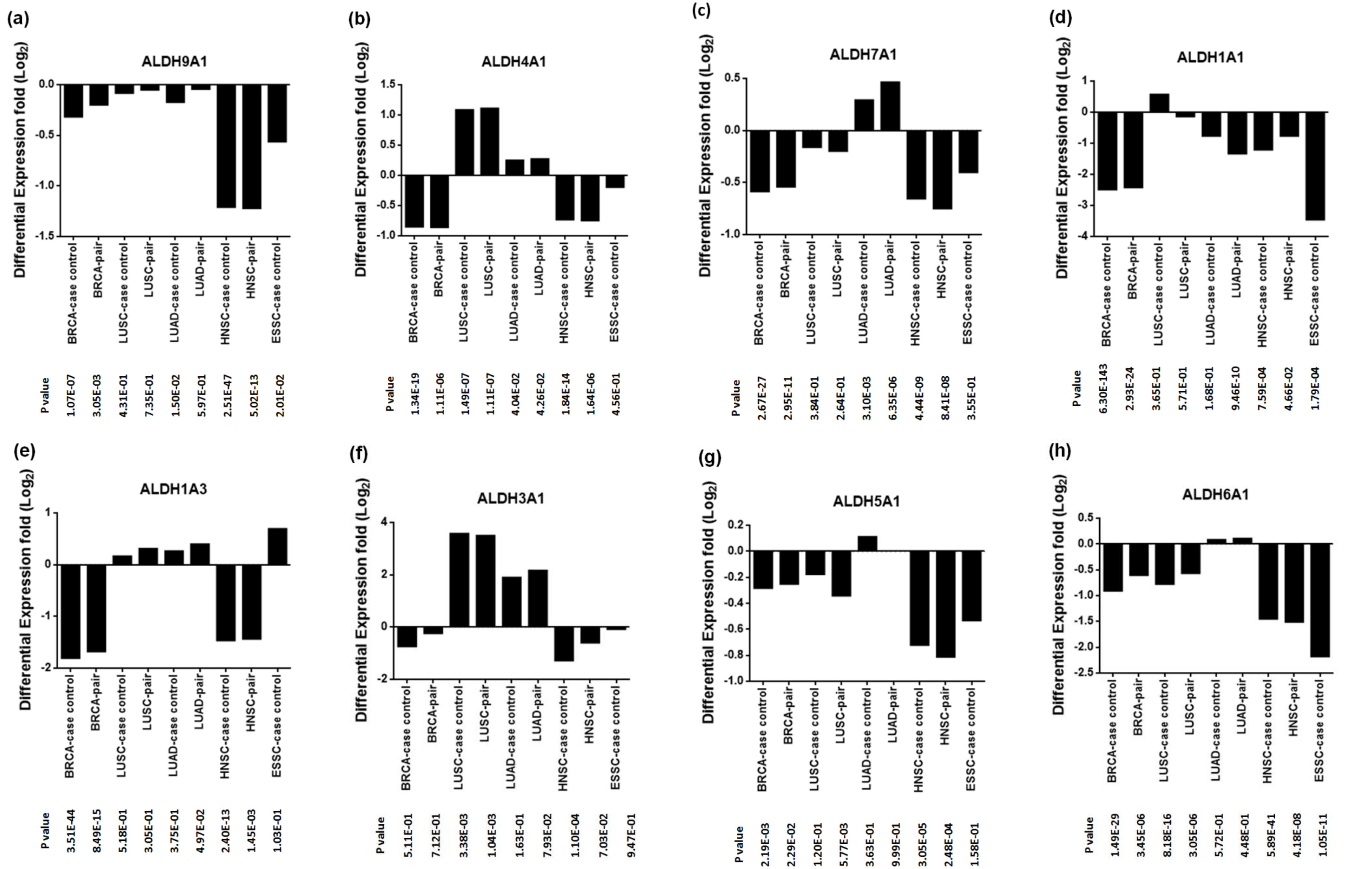
(a)
ALDH9A1
Differential Expression fold (Log2)
0.0
-0.5
-1.0
-1.5
BRCA-case control
BRCA-pair
LUSC-case control
LUSC-pair
LUAD-case control
LUAD-pair
HNSC-case control
HNSC-pair
ESSC-case control
P value
1.07E-07
3.05E-03
4.31E-01
7.35E-01
1.50E-02
5.97E-01
2.51E-47
5.02E-13
2.01E-02
(b)
ALDH4A1
Differential Expression fold (Log2)
1.5
1.0
0.5
0.0
-0.5
-1.0
BRCA-case control
BRCA-pair
LUSC-case control
LUSC-pair
LUAD-case control
LUAD-pair
HNSC-case control
HNSC-pair
ESSC-case control
P value
1.34E-19
1.11E-06
1.49E-07
1.11E-07
4.04E-02
4.26E-02
1.84E-14
1.64E-06
4.56E-01
(c)
ALDH7A1
Differential Expression fold (Log2)
0.5
0.0
-0.5
-1.0
BRCA-case control
BRCA-pair
LUSC-case control
LUSC-pair
LUAD-case control
LUAD-pair
HNSC-case control
HNSC-pair
ESSC-case control
P value
2.67E-27
2.95E-11
3.84E-01
2.64E-01
3.10E-03
6.35E-06
4.44E-09
8.41E-08
3.55E-01
(d)
ALDH1A1
Differential Expression fold (Log2)
1
0
-1
-2
-3
-4
BRCA-case control
BRCA-pair
LUSC-case control
LUSC-pair
LUAD-case control
LUAD-pair
HNSC-case control
HNSC-pair
ESSC-case control
P value
6.30E-143
2.93E-24
3.65E-01
5.71E-01
1.68E-01
9.46E-10
7.59E-04
4.66E-02
1.79E-04
(e)
ALDH1A3
Differential Expression fold (Log2)
1
0
-1
-2
BRCA-case control
BRCA-pair
LUSC-case control
LUSC-pair
LUAD-case control
LUAD-pair
HNSC-case control
HNSC-pair
ESSC-case control
P value
3.51E-44
8.49E-15
5.18E-01
3.05E-01
3.75E-01
4.97E-02
2.40E-13
1.45E-03
1.03E-01
(f)
ALDH3A1
Differential Expression fold (Log2)
4
2
0
-2
BRCA-case control
BRCA-pair
LUSC-case control
LUSC-pair
LUAD-case control
LUAD-pair
HNSC-case control
HNSC-pair
ESSC-case control
P value
5.11E-01
7.12E-01
3.38E-03
1.04E-03
1.63E-01
7.93E-02
1.10E-04
7.03E-02
9.47E-01
(g)
ALDH5A1
Differential Expression fold (Log2)
0.2
0.0
-0.2
-0.4
-0.6
-0.8
-1.0
BRCA-case control
BRCA-pair
LUSC-case control
LUSC-pair
LUAD-case control
LUAD-pair
HNSC-case control
HNSC-pair
ESSC-case control
P value
2.19E-03
2.29E-02
1.20E-01
5.77E-03
3.63E-01
9.99E-01
3.05E-05
2.48E-04
1.58E-01
(h)
ALDH6A1
Differential Expression fold (Log2)
0.5
0.0
-0.5
-1.0
-1.5
-2.0
-2.5
BRCA-case control
BRCA-pair
LUSC-case control
LUSC-pair
LUAD-case control
LUAD-pair
HNSC-case control
HNSC-pair
ESSC-case control
P value
1.49E-29
3.45E-06
8.18E-16
3.05E-06
5.72E-01
4.48E-01
5.89E-41
4.18E-08
1.05E-11

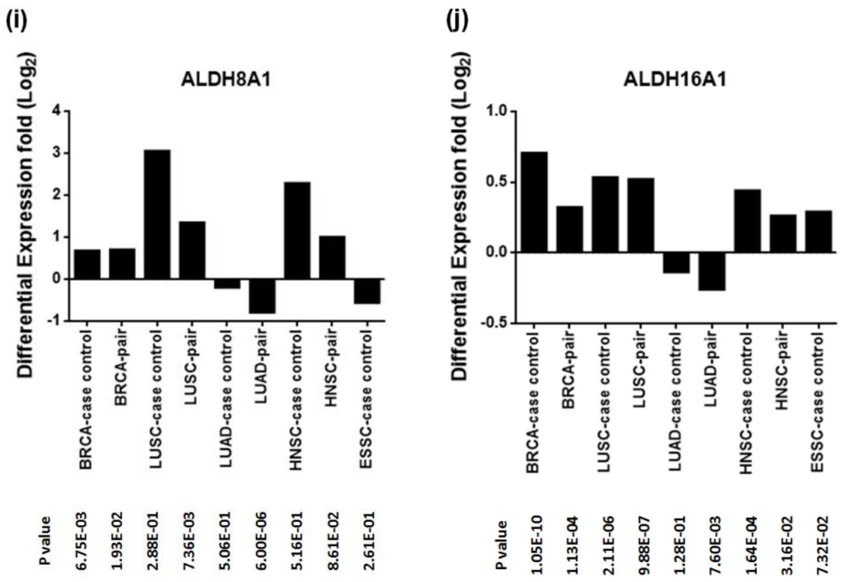

Suppl. Figure 1. Various differential expression of ALDH isoforms: (a) ALDH9A1 (b) ALDH4A1 (c) ALDH7A1 (d) ALDH1A1 (e) ALDH1A3 (f) ALDH3A1 (g) ALDH5A1 (h) ALDH6A1 (i) ALDH8A1 (j) ALDH16A1. Column from left to right: BRCA-casecontrol, BRCA-pair, LUSC-casecontrol, LUSC-pair, LUAD-casecontrol, LUAD-pair, HNSC-casecontrol, HNSC-pair, ESSC-casecontrol.

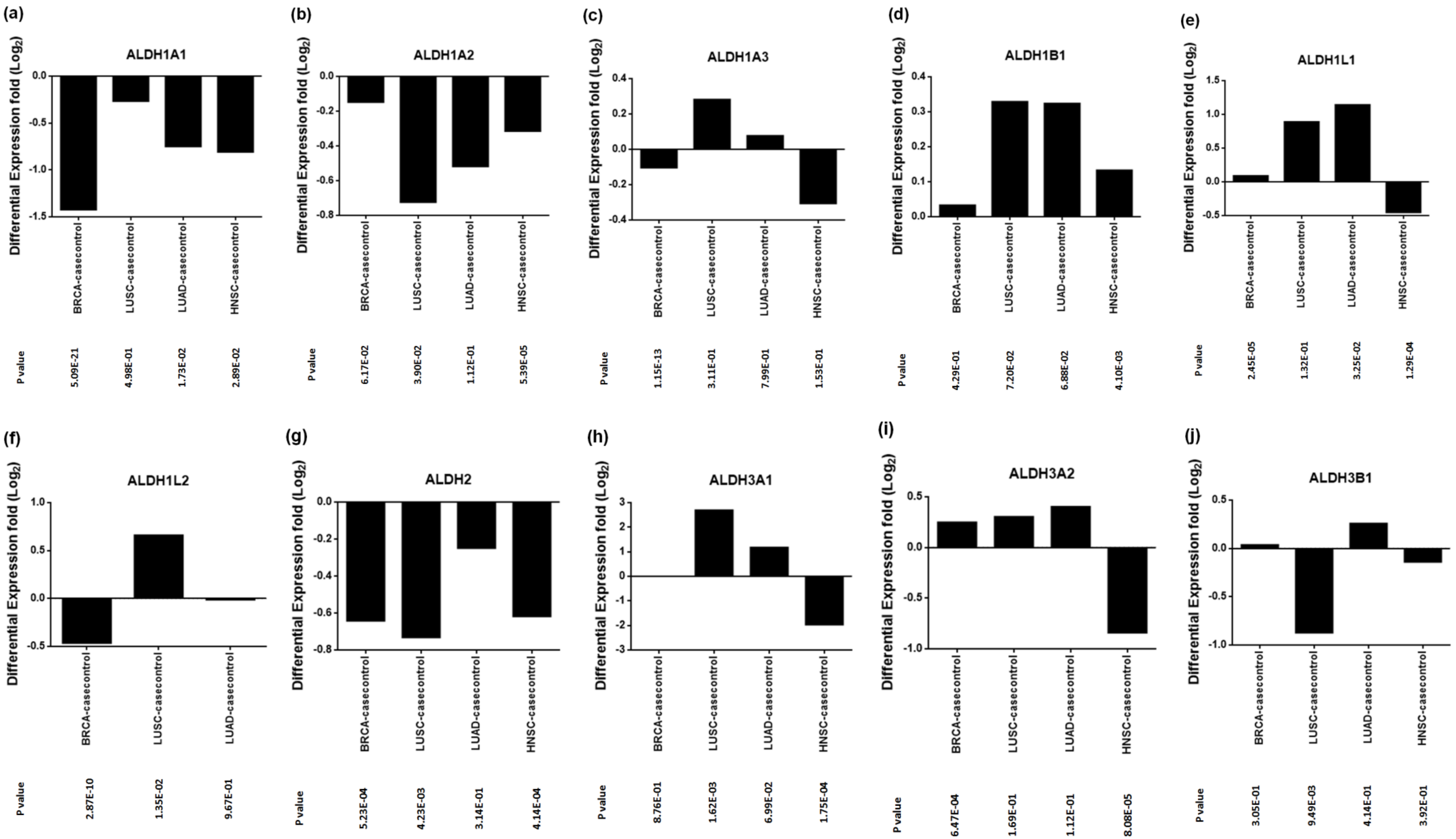

(a)
ALDH1A1
Differential Expression fold (Log2)
0.0
-0.5
-1.0
-1.5
BRCA-casecontrol
LUSC-casecontrol
LUAD-casecontrol
HNSC-casecontrol
P value
5.09E-21
4.98E-01
1.73E-02
2.89E-02
(b)
ALDH1A2
Differential Expression fold (Log2)
0.0
-0.2
-0.4
-0.6
-0.8
BRCA-casecontrol
LUSC-casecontrol
LUAD-casecontrol
HNSC-casecontrol
P value
6.17E-02
3.90E-02
1.12E-01
5.39E-05
(c)
ALDH1A3
Differential Expression fold (Log2)
0.4
0.2
0.0
-0.2
-0.4
BRCA-casecontrol
LUSC-casecontrol
LUAD-casecontrol
HNSC-casecontrol
P value
1.15E-13
3.11E-01
7.99E-01
1.53E-01
(d)
ALDH1B1
Differential Expression fold (Log2)
0.4
0.3
0.2
0.1
0.0
BRCA-casecontrol
LUSC-casecontrol
LUAD-casecontrol
HNSC-casecontrol
P value
4.29E-01
7.20E-02
6.88E-02
4.10E-03
(e)
ALDH1L1
Differential Expression fold (Log2)
1.5
1.0
0.5
0.0
-0.5
BRCA-casecontrol
LUSC-casecontrol
LUAD-casecontrol
HNSC-casecontrol
P value
2.45E-05
1.32E-01
3.25E-02
1.29E-04
(f)
ALDH1L2
Differential Expression fold (Log2)
1.0
0.5
0.0
-0.5
BRCA-casecontrol
LUSC-casecontrol
LUAD-casecontrol
P value
2.87E-10
1.35E-02
9.67E-01
(g)
ALDH2
Differential Expression fold (Log2)
0.0
-0.2
-0.4
-0.6
-0.8
BRCA-casecontrol
LUSC-casecontrol
LUAD-casecontrol
HNSC-casecontrol
P value
5.23E-04
4.23E-03
3.14E-01
4.14E-04
(h)
ALDH3A1
Differential Expression fold (Log2)
3
2
1
0
-1
-2
-3
BRCA-casecontrol
LUSC-casecontrol
LUAD-casecontrol
HNSC-casecontrol
P value
8.76E-01
1.62E-03
6.99E-02
1.75E-04
(i)
ALDH3A2
Differential Expression fold (Log2)
0.5
0.0
-0.5
-1.0
BRCA-casecontrol
LUSC-casecontrol
LUAD-casecontrol
HNSC-casecontrol
P value
6.47E-04
1.69E-01
1.12E-01
8.08E-05
(j)
ALDH3B1
Differential Expression fold (Log2)
0.5
0.0
-0.5
-1.0
BRCA-casecontrol
LUSC-casecontrol
LUAD-casecontrol
HNSC-casecontrol
P value
3.05E-01
9.49E-03
4.14E-01
3.92E-01

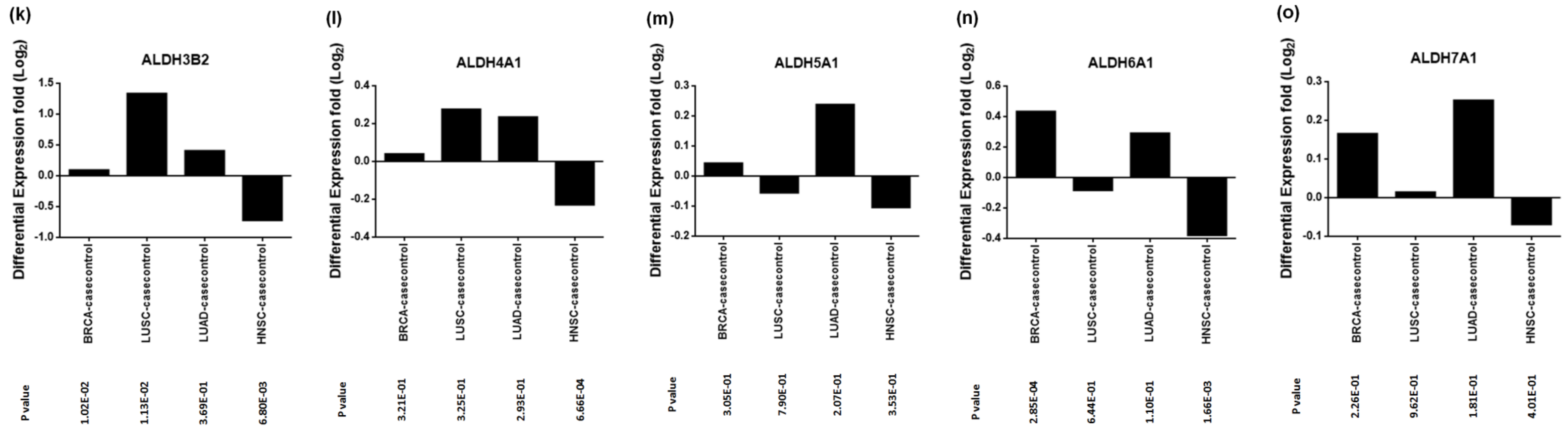

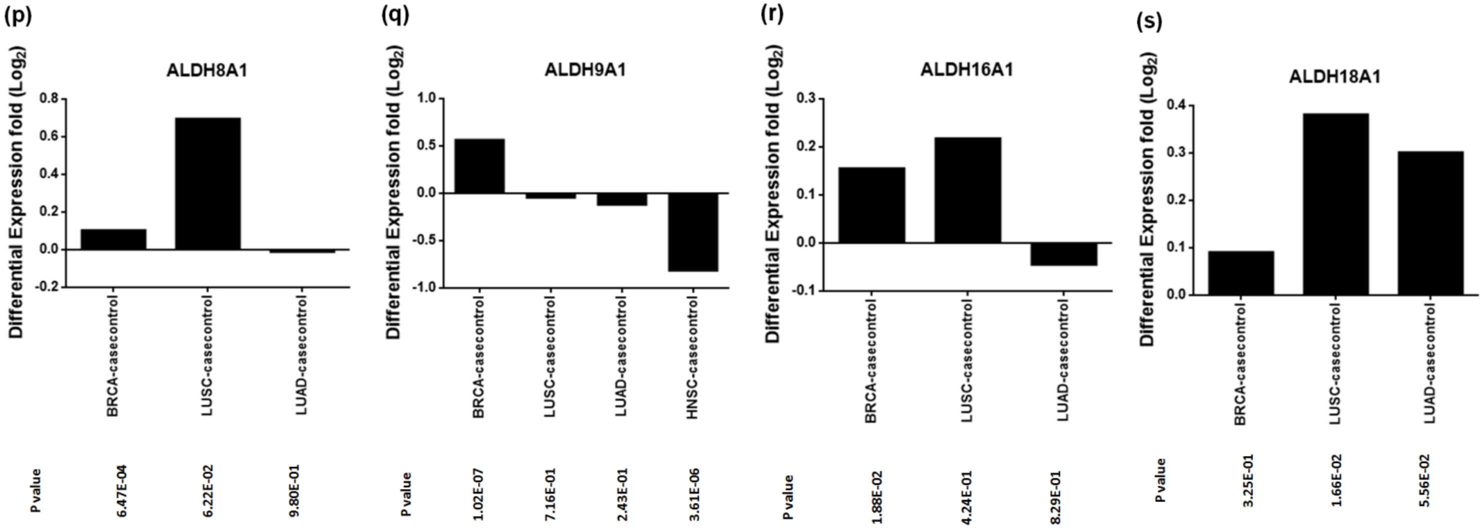

**Suppl. Figure 2. Validation cohorts from other non-TCGA data. ***There were no ALDH1L2, ALDH8A1, ALDH16A1, and ALDH18A1 probsets in HNSC microarray profile (GSE6631).

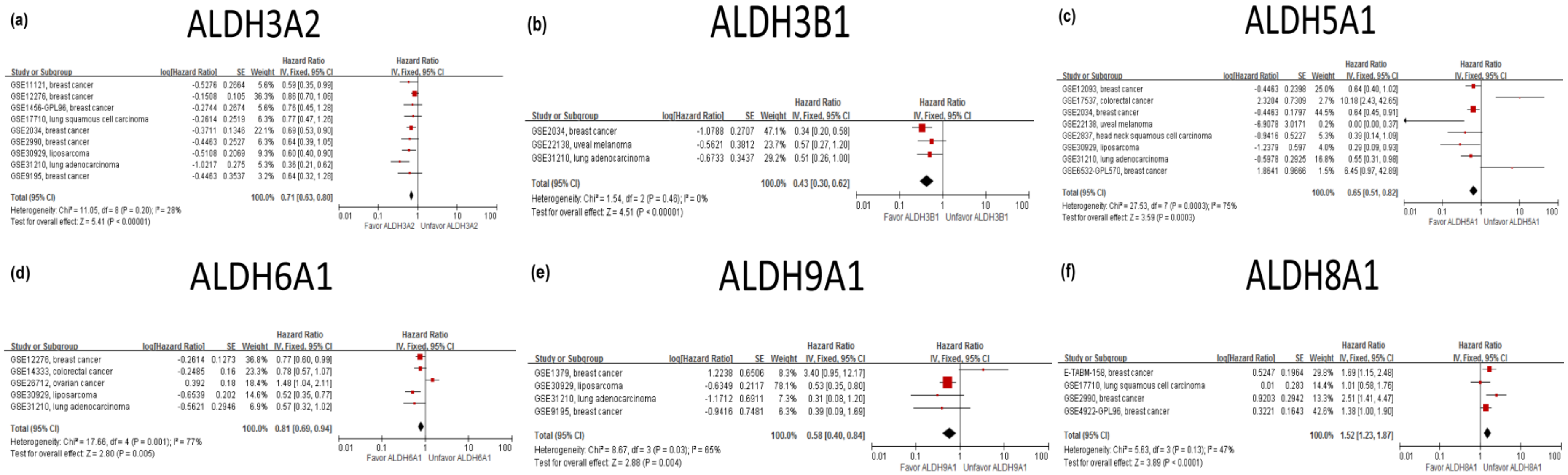

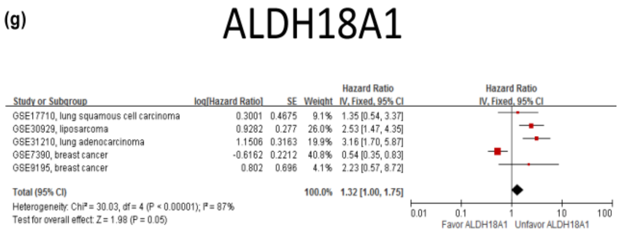

Suppl. Figure 3. Meta-analysis for separated progression free survival in different ALDH isoforms. "Favor" means expression of candidate ALDH toward better prognosis, while "Unfavor" means expression toward poorer prognosis. All meta-analysis run in fixed module.

Suppl. Table 1. Differential expression of ALDH2 in HNSC from VGHTPE cohort

Gene Name	Gene ID	Gene Description	Gene Type	Comparison	Normal	Tumor	fold change* (log2)	p-value
ALDH2	217	aldehyde dehydrogenase 2 family (mitochondrial)	protein-coding	Pairwise	69.3334	55.4675	-0.321909040337392	0.55162
ALDH2	217	aldehyde dehydrogenase 2 family (mitochondrial)	protein-coding	Case-control	69.3334	47.2564	-0.553040685539472	0.27023
EGFR	1956	epidermal growth factor receptor	protein-coding	Pairwise	15.6005	23.9695	0.619612113675511	0.20773
EGFR	1956	epidermal growth factor receptor	protein-coding	Case-control	15.6005	32.6247	1.0643776278144	0.16292

*tumor/normal (log2)

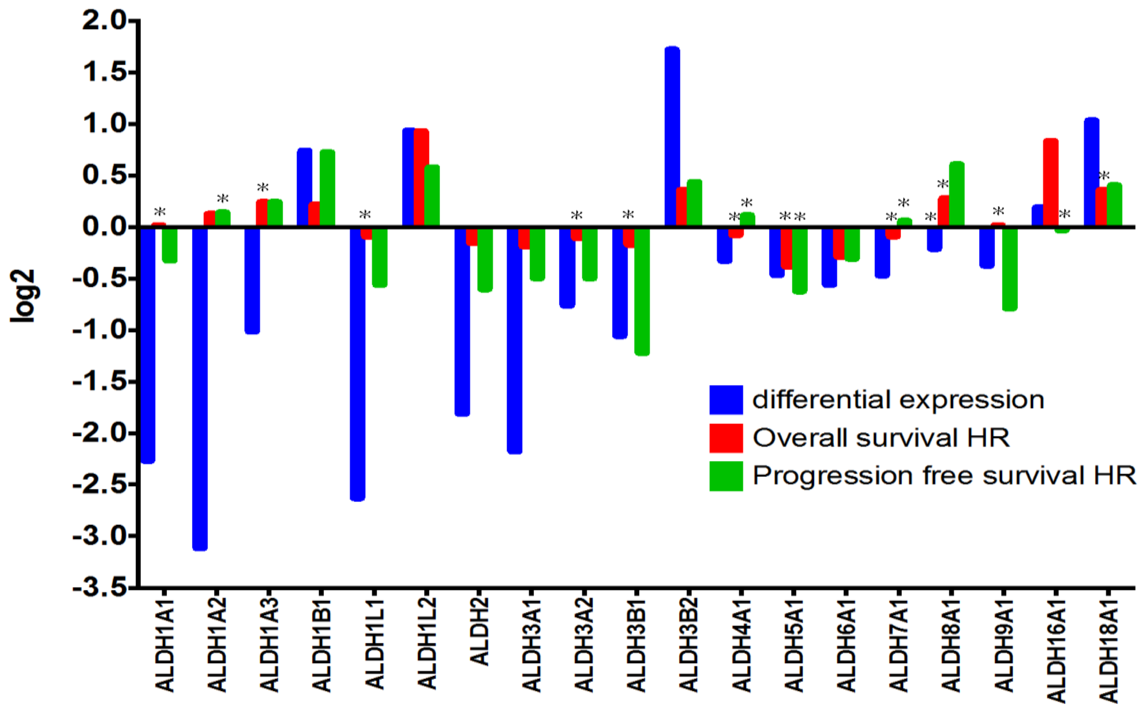

Suppl. Figure 4. ALDH differential expression has consensus with prognosis. All expression and hazard ratio were shown in log_2 transformation. All p value is less than 0.05 except marked with asterisk (*). HR: hazard ratio.

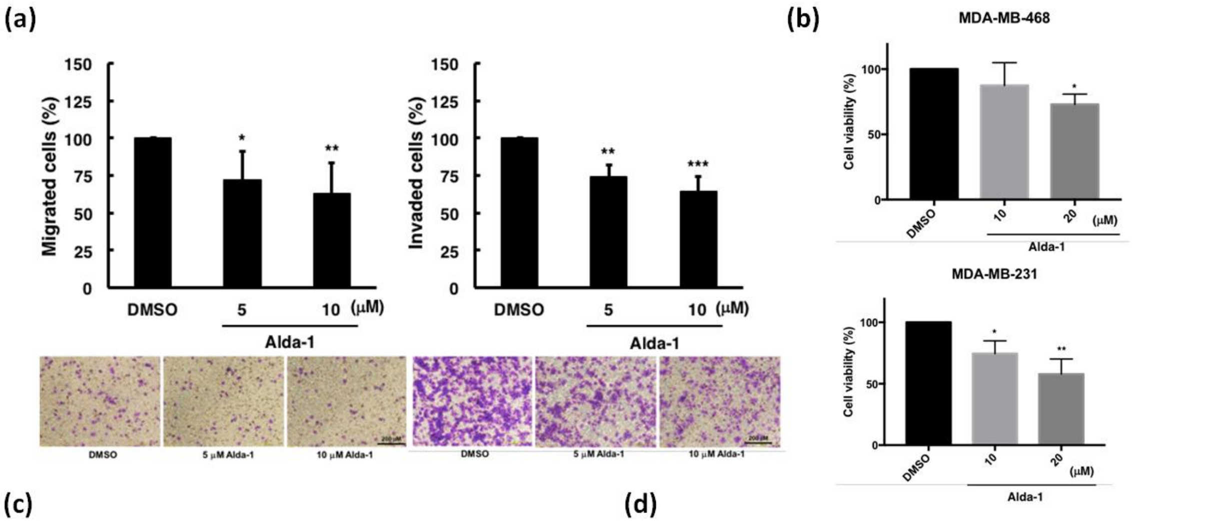

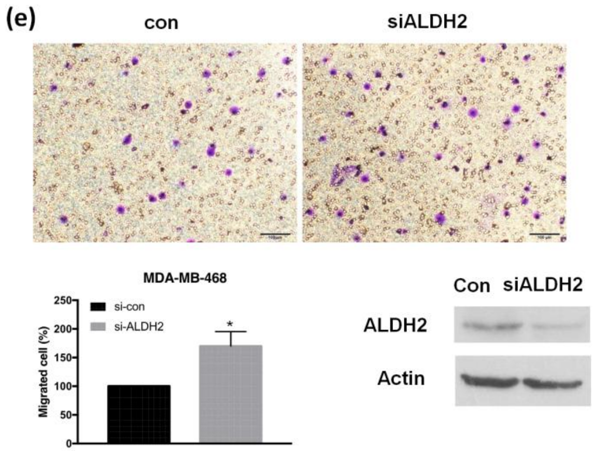

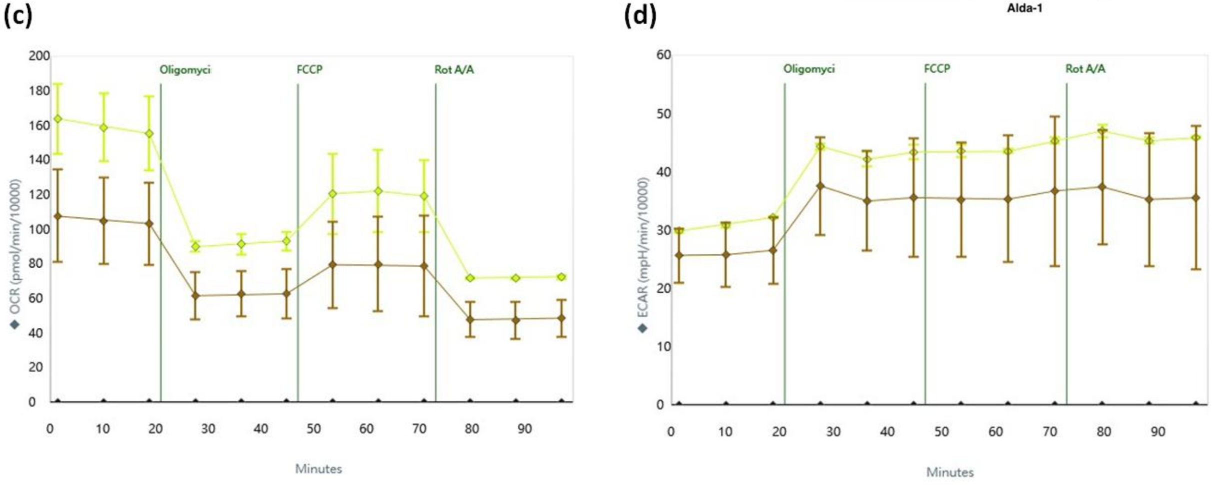

Suppl. Figure 5. *In vitro* modulating ALDH2 activity influence caner phenotypes. (a) Migration/Invasion assays showed dose dependently inhibition by ALDH2 agonist, Alda-1, in MDA-MB-468 cells. (b) Alda-1 inhibited MDA-MB-468 and MDA-MB-231 cells viability especially over 10uM. (c) Alda-1 inhibited oxidation consumption rate (OCR) of FaDu cells. Green line: control; Brown line: with Alda-1 treatment. (d) Alda-1 inhibited extra cellular acidification rate (ECAR) in FaDu cells. Green line: control; Brown line: with Alda-1 treatment. (e) knockdown ALDH2 with siRNA showed increased cell migration ability in MDA-MB-468 cells. * $p < 0.05$, ** $p < 0.01$, *** $p < 0.001$.

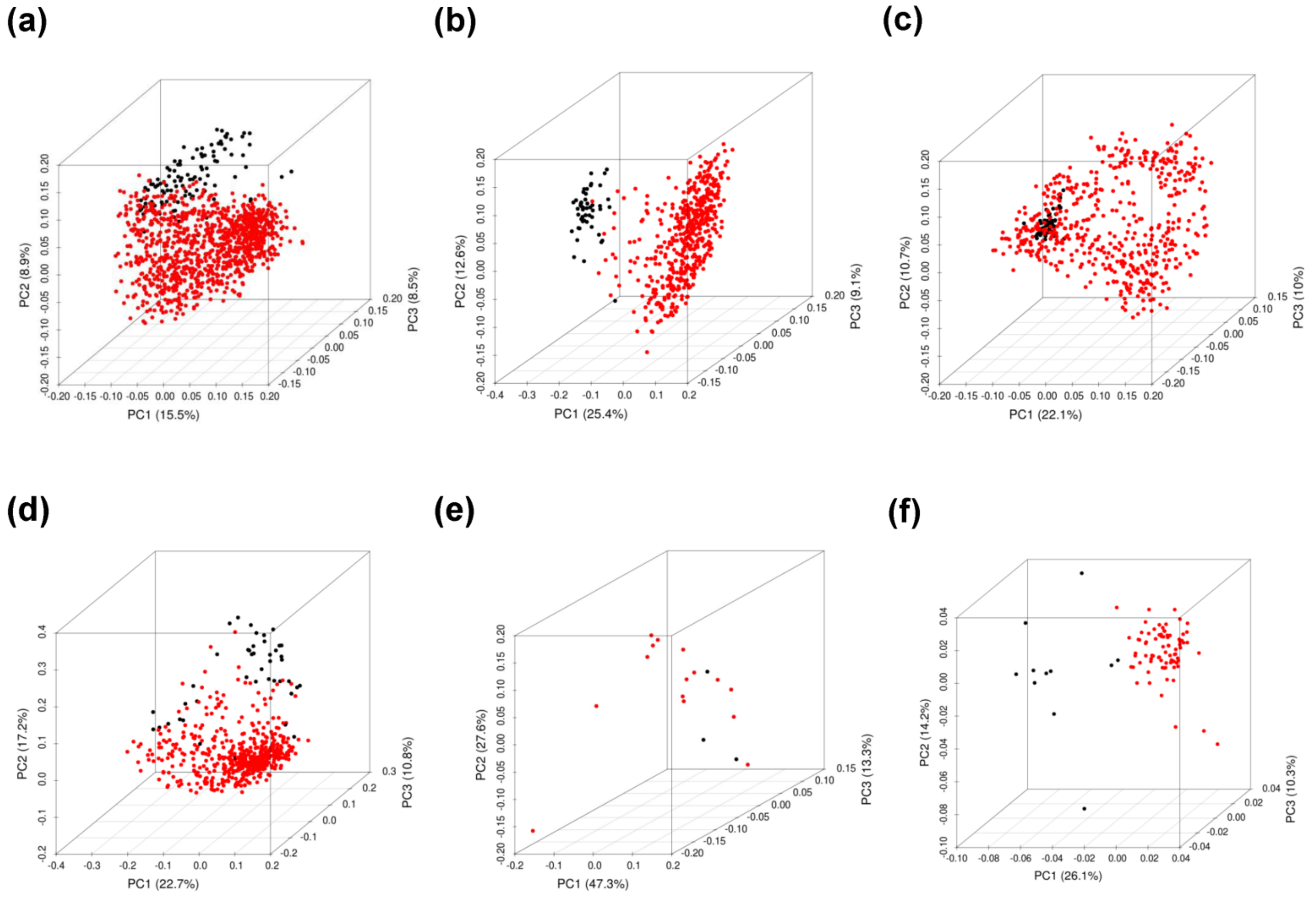

Suppl. Figure 6. PCA plot of gene expression comparison between tumor (red) vs. non-tumor (black). (a) breast cancer (b) lung squamous cell carcinoma (c) lung adenocarcinoma (d) head and neck squamous cell carcinoma. (e) head and neck squamous cell carcinoma from VGHTPE cohort. (f) esophageal squamous cell carcinoma. Each point on the 3-D plot represents a sample and distance between every two points represents level of similarity of these two points. The distribution for VGHTPE (e) and TCGA head and neck squamous cell carcinoma (d) both showed higher individual dispersion between tumor vs. non-tumor tissues
